# Chronic Phencyclidine Increases Synapsin-1 and Synaptic Adaptation Proteins in the Medial Prefrontal Cortex

**DOI:** 10.1155/2013/620361

**Published:** 2013-02-19

**Authors:** Chris Pickering, Mia Ericson, Bo Söderpalm

**Affiliations:** ^1^Addiction Biology Unit, Department of Psychiatry and Neurochemistry, Institute of Neuroscience and Physiology, University of Gothenburg, P.O. Box 410, 405 30 Gothenburg, Sweden; ^2^Beroendekliniken, Sahlgrenska University Hospital, Gothenburg, Sweden

## Abstract

Phencyclidine (PCP) mimics many aspects of schizophrenia, yet the underlying mechanism of neurochemical adaptation for PCP is unknown. We therefore used proteomics to study changes in the medial prefrontal cortex in animals with PCP-induced behavioural deficits. Male Wistar rats were injected with saline or 5 mg/kg phencyclidine for 5 days followed by two days of washout. Spontaneous alternation behaviour was tested in a Y-maze and then proteins were extracted from the medial prefrontal cortex. 2D-DIGE analysis followed by spot picking and protein identification with mass spectrometry then provided a list of differentially expressed proteins. Treatment with 5 mg/kg phencyclidine decreased the percentage of correct alternations in the Y-maze compared to saline-treated controls. Proteomics analysis of the medial prefrontal cortex found upregulation of 6 proteins (synapsin-1, Dpysl3, Aco2, Fscn1, Tuba1c, and Mapk1) and downregulation of 11 (Bin1, Dpysl2, Sugt1, ApoE, Psme1, ERp29, Pgam1, Uchl1, Ndufv2, Pcmt1, and Vdac1). A trend to upregulation was observed for Gnb4 and Capza2, while downregulation trends were noted for alpha-enolase and Fh. Many of the hits in this study concur with recent postmortem data from schizophrenic patients and this further validates the use of phencyclidine in preclinical translational research.

## 1. Introduction

Schizophrenia is a complex, relapsing psychiatric disorder featuring both positive and negative symptoms. Developing medication to treat schizophrenia has been difficult, and various animal models have been proposed to aid the search for treatments. For example, the MATRICS battery of tests is now used to screen the efficacy of novel compounds [1] and groups such as Bussey and colleagues are continually optimizing these methods for use on a larger scale or by industry [2]. One can mimic the symptoms of schizophrenia clinically with the unselective compound phencyclidine (PCP) [3]. Repeated injection of PCP in animals induces dysfunction of the prefrontal cortex which can be measured both behaviourally and neurochemically [4]. However, comparison of these results is complicated by the multitude of injection schedules and protocols depending on animal (e.g., rat versus mouse) or strain in question [5–10]. 

Given the diversity of phencyclidine protocols, it is important to validate our results with others and with the clinical situation. The underlying neurochemical changes induced by PCP in the medial prefrontal cortex are still not well understood. Most methods have focused on metabolic measures (e.g., glucose utilization) [10], proposed changes in GABAergic interneurons (e.g., parvalbumin) [4], or dendritic spine density [11], but little has been studied in terms of large-scale changes in protein expression. In a recent review of proteomic studies of postmortem brains of schizophrenics, a list of differentially expressed proteins is provided [12]. Inclusion criteria were frontal brain areas (6 dorsolateral PFC, 1 anterior cingulate, and 1 insular cortex) and that the protein was significant in at least two studies. In order to validate our PCP model as a representation of synaptic changes, we have investigated differences in protein expression between PCP and saline-treated animals using the 2D-DIGE method followed by protein identification using mass spectrometry. We then compared our results to the review of English and colleagues [12] to validate this model to represent the schizophrenic individual.

## 2. Materials and Methods

A total of 8 naïve male Wistar rats with weight of 190–210 g upon arrival were used in this study (age corresponds to adolescence during injection). Animals were housed four per cage in a temperature- and humidity-controlled animal care facility (lights on at 07.00, 12-hour light : dark cycle) and were allowed to acclimatize to the environment for one week before the commencement of the study. All experiments were approved by the Ethical Committee for Use of Animal Subjects (DNr 48/11). Animal care procedures followed the guidelines of the Swedish legislation on animal experimentation (Animal Welfare Act SFS1998:56) and the EU legislation (Convention ETS123 and Directive 86/609/EEC).

### 2.1. Testing Protocol

Animals were divided into two groups: saline (*n* = 4) and 5 mg/kg PCP (*n* = 4) group. Phencyclidine hydrochloride (Sigma-Aldrich, Stockholm, Sweden) was dissolved in 0.9% NaCl and injected intraperitoneal (i.p.). Injections occurred once per day at approximately 13.00 from Monday to Friday followed by the weekend without handling or drug administration. On Monday, animals were tested in the Y-maze (see below).

The Y-maze contains three arms made out of nonreflective grey plastic which fit together in a grooved, metal, nonreflective base. The dimensions of the arms were 50 cm long, 10 cm wide, and 20 cm high walls (Stoelting Europe). Movement was automatically tracked using the ANY-maze software via a camera mounted directly above the maze. No habituation sessions were provided although animals were handled previously in the testing room. The animal was placed in an arm facing the centre (Arm A) and its free exploration was recorded for 10 min. A correct alternation occurred when the animal entered the other two arms without retracing its steps (i.e., Arm A to B to C). Persistent movements such as ABA were incorrect. Based on the arm entries over the entire session, the percentage of correct alternations was calculated. Differences between PCP and saline groups were determined using *t*-test or ANOVA when appropriate and *P* < 0.05 was considered significant. Occasionally, an animal would move to another arm and remain immobile for an extended period of time before movement to the third arm. To prevent misguiding results, that particular alternation was excluded if an animal remained immobile for more than 2 min. 

### 2.2. Sample Preparation

Upon completion of this test, the individual animal was taken to a separate room and was decapitated directly. The medial prefrontal cortex was dissected according to the Paxinos rat brain atlas [13]. Samples were flash-frozen on a dry ice block and stored at −80°C until further preparation. To separate total protein from the samples, the Qiagen DNA/RNA/Protein kit was used according to standard protocols. Protein pellets in ethanol were stored at −20°C and transported to the Proteomics Core Facility where the proteins were dissolved in buffer containing 7 M urea, 2 M thiourea, 4% CHAPS, and 30 mM Tris-HCl, pH 8.5.

### 2.3. Two-Dimensional Electrophoresis and Image Analysis

Protein expression in the medial prefrontal cortex of phencyclidine-treated animals (*n* = 4) was compared to saline-treated controls (*n* = 4). 2D-DIGE analysis was performed across 4 gels using the same pooled-sample internal standard and the equimolecular mixture of all the samples in each [14]. Samples were G-Dye labeled according to the manufacturer's standard protocol (NH DyeAGNOSTICS) using 400 pmol of dye reagent for 50 *μ*g of sample protein. Individual samples were labeled with G200 or G300 dyes using dye switching, and the internal standard was always G100-labeled. 

Isoelectric focusing (IEF) was performed in 24 cm pH 3–11 Nonlinear Imobiline DryStrips (GE Healthcare) on an Ettan IPGphor. The second dimension was run on an Ettan DALT II using in-house made 1 mm polyacrylamide (*T* = 11%, *C* = 2.6%) Bis-Tris gel with standard MOPS cathode buffer and acetic acid/diethanol amine anode buffer. After 2D electrophoresis, gels were scanned by the VersaDoc MP 4000 (Bio-Rad) using the excitation/emission wavelengths specific for the different G-Dyes. Gel images were analysed using the Progenesis SameSpots software ver. 4.1 (nonlinear dynamics) for spot detection, spot quantification, intergel matching, and statistics (ANOVA). This software also takes multiple comparisons into account given the repeated use of ANOVA to compare each protein. Spots were then selected for spot picking and further identification by MS analysis. Gel spots were considered statistically significant at *P* < 0.05, while trends towards significance included *P* < 0.10.

### 2.4. Spot Picking and In-Gel Protein Digestion

Selected protein spots of interest were picked and trypsinated in the Ettan Spot Handling Workstation (GE Healthcare). Spots were taken from a separate preparative gel of pooled samples with a combined total protein concentration of 450 *μ*g stained with RuBPS (ruthenium(II) tris(bathophenanthroline disulfonate), RubiLAB, Switzerland). The method for in-gel protein digestion with trypsin described by Shevchenko and colleagues was applied with some minor modifications [15]. Briefly, the gel pieces were destained by washing three times in 25 mM NH_4_HCO_3_ in 50% CH_3_OH and once in 70% CH_3_CN. Gel pieces were dried and incubated with digestion buffer (50 mM NH_4_HCO_3_, 10 ng/*μ*L trypsin) at 37°C for 3 h. Peptides were extracted in 50% CH_3_CN/0.5% TFA and the supernatant was evaporated to dryness. Prior to MS analysis, the peptides were reconstituted in 0.2% HCOOH until they are dry.

### 2.5. Protein Identification

For protein identification the minimum criteria were one tryptic peptide matched at or above the 99% level of confidence and one additional peptide match at the 95% level.

#### 2.5.1. LC-MS/MS

Sample injections were made with an HTC-PAL autosampler (CTC Analytics AG, Zwingen, Switzerland) connected to an Agilent 1100 binary pump (Agilent Technologies, Palo Alto, CA, USA). The peptides were trapped on a precolumn (45 × 0.075 mm i.d.) and separated on a reversed-phase column, 200 × 0.050 mm. Both columns are packed in-house with 3 *μ*m ReproSil-Pur C18-AQ particles. The flow-through to the analytical column was reduced by a split of approximately 100 nL/min. A 40 min gradient of 10–50% CH_3_CN in 0.2% COOH was used for separation of the peptides. For complete details, see [16].

The nanoflow LC-MS/MS was performed on a hybrid linear ion trap-FTICR mass spectrometer equipped with a 7 T ICR magnet (LTQ-FT, Thermo Electron, Bremen, Germany). The spectrometer was operated in a data-dependent mode, automatically switching to the MS/MS mode. MS spectra were acquired in the FTICR, while MS/MS spectra were acquired in the LTQ-trap. For each scan of FTICR, the three most intense, doubly or triply charged, ions were sequentially fragmented in the linear trap by collision-induced dissociation. All the tandem mass spectra were searched against mammals in the NCBI database by MASCOT software (Matrix Science, London, UK). The search parameters were set to MS accuracy 5 ppm, MS/MS accuracy 0.5 Da, one missed cleavage by trypsin allowed, fixed propionamide modification of cysteine, and variable modification of oxidized methionine.

#### 2.5.2. MALDI TOF MS/MS

Selected samples were analyzed on a MALDI TOF/TOF (ultrafleXtreme, Bruker Daltonics, Bremen, Germany) instrument operated in the positive ion mode. The analyzed mass range was 700–4000 Da with ion suppression up to 600 Da. MS and MS/MS analyses were performed automatically. For MS analysis, 2000 single-shot spectra were accumulated by recording 200-shot spectra at 10 random positions using fixed laser attenuation. Selection of precursor ions for MS/MS was performed using the Warp-LC software (Bruker Daltonics). For MS/MS analysis, 2000 single shots spectra were recorded for the precursors and 4000 for the fragment ion spectra. Peptide and tandem mass spectra were searched against mammals in the Swiss-Prot database by the MASCOT software. The search parameters were set to MS accuracy 50 ppm, MS/MS accuracy 0.7 Da, one missed cleavage by trypsin allowed, fixed propionamide modification of cysteine, and variable modification of oxidized methionine.

### 2.6. In Silico Analysis

To study the relationship between proteins significantly affected by PCP treatment, the names of the genes were entered into the String Database 9.0 (http://string-db.org/). Evidence for functional connections, coexpression, interactions, and experimental results was then reviewed to see whether our experimental evidence corresponds to what is known. 

## 3. Results

### 3.1. Behaviour Analysis

**Figure 1:**
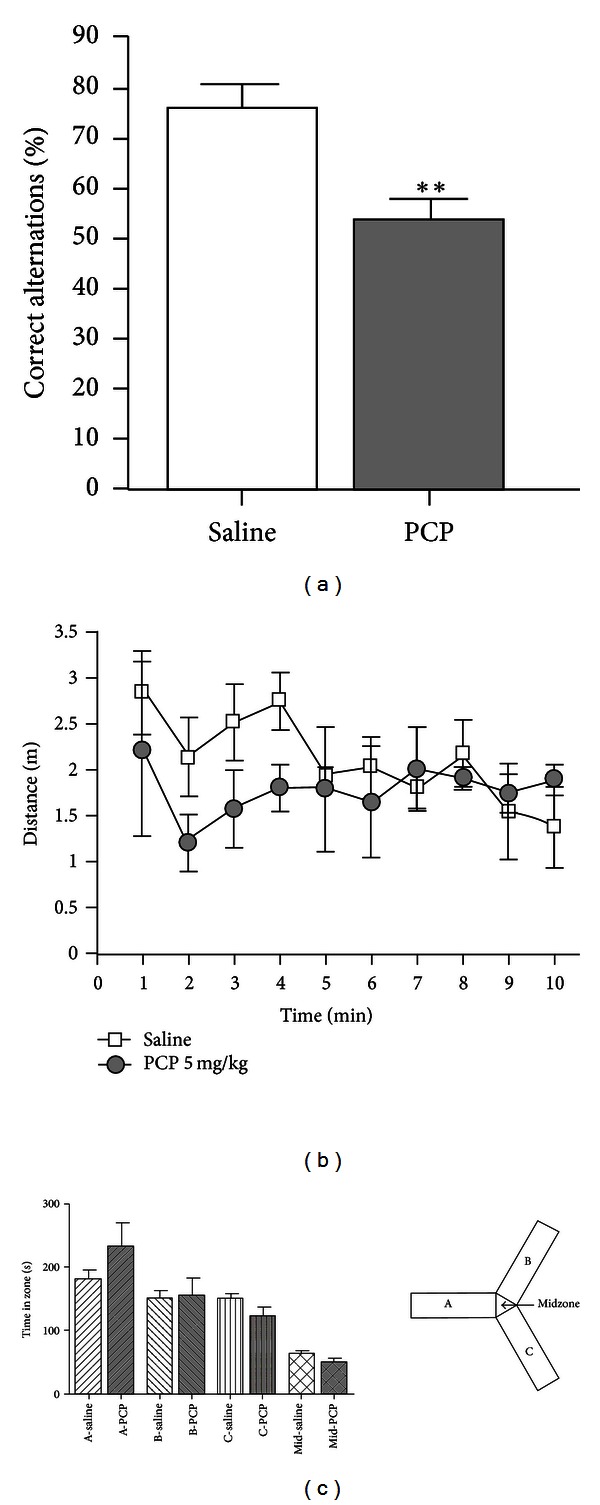
Behavioural characterization of animals treated with saline or phencyclidine (PCP) for 5 days followed by 2 days of washout. (a) PCP treatment significantly decreased the percentage of correct alternations. **P < 0.01. (b) Distance moved across the 10 min session. For the first 5 min, PCP animals moved significantly less than saline animals, but there was no group difference for the second half of the session. (c) Groups did not show any differential preference for a particular arm or zone of the Y-maze.

A comparison of the behaviour of animals receiving saline (*n* = 4) or PCP (*n* = 4) is illustrated in Figure 1. The main outcome variable for prefrontal cortex function was the percentage of correct alternations (Figure 1(a)). PCP-treated animals made more incorrect alternations compared to controls (*t*-test, *P* = 0.0089). The total distance moved by the animals in the Y-maze can explain the time course of the PFC dysfunction (Figure 1(b)). A 2-way ANOVA of the first 5 min of the session found a significant decrease in total distance in the PCP-treated animals (factor drug: *F* = 5.43, *P* = 0.027) but a consistent movement over time (factor time: *F* = 0.96, *P* = 0.44). This indicates a measurable effect of PCP during the first 5 min but no difference compared to controls for the remaining 5 min of the 10 min session. In terms of preference for a given arm of the Y-maze (Figure 1(c)), animals did not prefer a certain arm and there were no differences between PCP or saline groups. The apparent difference between treatment groups in Zone A was not significant (*t*-test, *P* = 0.24).

### 3.2. Proteomics

After protein extraction, samples were labeled with fluorescence and run on a 2D gel and the difference in expression of each protein spot was quantified and compared by ANOVA. Proteins were identified in the spots significantly different using one of two mass spectrometry methods. Identified proteins are listed in Table 1. In total, 6 proteins were upregulated by PCP in the prefrontal cortex and two had a trend to significance (*P* < 0.10). Analysis found 11 proteins downregulated by PCP with an additional two with trend towards significance.

### 3.3. **In Silico** Analysis

**Figure 2:**
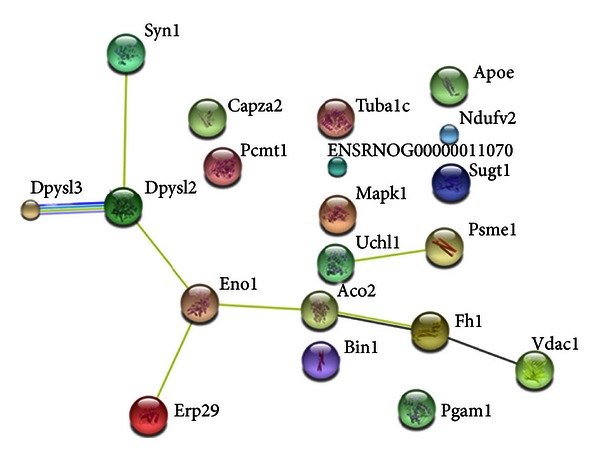
*In silico* analysis using the String 9.0 database of proteins significantly altered by phencyclidine. Synapsin-1 is associated with Dpysl2 and Dpysl3 which are in turn linked with ERp29. Coexpression studies link Fh1, Vdac1, and Aco2 together. Note: Fscn1 was not listed in the database.

To explore whether the proteins differentially affected by PCP treatment were related, an *in silico* analysis was made using the String Database 9.0. In Figure 2, possible connections between the different proteins are indicated. Synapsin-1 was linked with Dpysl2 (association score = 0.481), which was subsequently connected to Dpysl3 (association score = 0.904). This cluster was linked, via alpha-enolase (association score = 0.515) to ERp29 (association score = 0.562) and Aco2 (association score = 0.609). Aco2, Fh (association score = 0.662), and Vdac1 (association score = 0.581) were linked via coexpression data. Uchl1 and Psme1 (association score = 0.540) form an additional linked cluster apart from the others. Fscn1 was not listed in the rat database and was therefore excluded from the analysis.

## 4. Discussion

In this study we applied a standard phencyclidine treatment protocol, produced a measureable behavioural deficit in spontaneous alternation behavior, and could provide evidence of neuroadaptation of this via proteomics analysis of the medial prefrontal cortex. Proteins significantly affected by phencyclidine suggest that this drug changes synaptic transmission and plasticity and also affects glycolysis and gluconeogenesis [12]. Additionally, several of the hits in this study have also appeared in clinical postmortem studies of the brains of individuals with schizophrenia, thus further validating the use of phencyclidine as a model of human disorder.

The significant upregulation of synapsin-1 protein by PCP has never been observed previously although other synapsins are related to schizophrenia. Synapsin-2 knockout mice display many of the behavioural deficits observed after PCP treatment [17], but this could be explained by an upregulation of synapsin-1 to counteract the loss of synapsin-2. A clinical report found a downregulation of synapsin-2 mRNA in the prefrontal cortex of schizophrenic patients [18], and the synapsin-2 knockdown in the medial prefrontal cortex of rats is discussed as an animal model of schizophrenia [19]. The findings in our study also suggest an involvement of synapsin-1 such that a more general investigation of the role of synapsins in schizophrenia is warranted. Synapsins are involved in the transport of synaptic vesicles to the active space [20] and an upregulation could be expected to increase synaptic transmission [21]. Previous studies of chronic PCP administration found an increase in dendritic spine density in pyramidal neurons of the medial prefrontal cortex [11] which also confirms an increase in synaptic transmission. 

Two of the dihydropyrimidinase-related (Dpysl) proteins were affected by PCP treatment and the link of these with synapsin-1 according to *in silico* analysis in the String 9.0 database supports the strength of these findings. Dpysl3 was upregulated by PCP while Dpysl2 was downregulated in the medial prefrontal cortex. Decreased Dpysl2 concurs with clinical studies and, together with our finding of decreased Uchl1, suggests altered axonal signaling and synaptic pruning [12]. Dpysl2 is the most abundant of this protein family and is involved in neuronal differentiation and axon guidance [22]. Dpysl2 is affected in schizophrenia, Alzheimer's disease, and Parkinson disease and is upregulated following brain ischemia [22, 23]. Less is known about Dpysl3 but one postmortem study of patients with Down syndrome noted an upregulation of Dypsl3 and downregulation of Dypsl2 [24], the same pattern we observed after PCP treatment. 

Aconitase (Aco2) is a key mitochondrial enzyme in the citric acid cycle which is active in the presence of iron [25]. In the absence of sufficient iron or during oxidative stress, aconitase binds to mRNA at iron-responsive elements [26] and is also thought to protect mitochondrial DNA from damage [27]. The role of oxidative stress in Parkinson disease progression is well established, but aconitase appears to conflict with this process [25]. The upregulation of the enzyme itself, irrespective of active state, by PCP is also difficult to explain but could reflect an iron-deficient state where neuroprotection is favoured over energy production.

The remaining proteins upregulated by PCP suggest adaptations to support the increased synaptic response. Mapk1, or extracellular signal regulated kinases-1/2 (ERK1/2), provides a signal transduction system in neurons which is able to respond rapidly to extracellular messages [28]. Mapk1 in its phosphorylated form accumulates in dying neurons in Alzheimer's disease [28] which indicates a risk for toxicity with prolonged PCP treatment. Fascin-1 (Fscn1), an actin cross-linking protein abundant in most brain cell types, is thought to anchor key components in the synaptic region [29] and is upregulated in an animal model of Down syndrome [30]. Similarly, the tubulin Tuba1c is involved in microtubule formation which is regulated by acetylation [31]. The regulation of vesicular trafficking and the promotion of the axonal transport of BDNF are also attributable to Tuba1c [31].

A total of 11 proteins were downregulated by repeated injections of PCP and these can be broadly placed into three classes. The first includes changes that will influence cellular energy production and subsequent neuronal activity. Phosphoglycerate mutase 1 (Pgam1) is an enzyme late in the glycolysis pathway converting 3-phosphoglycerate to 2-phosphoglycerate and decreases in this will lead to less pyruvate [32]. Clinical postmortem analyses have found less Pgam1 and also less thalamic pyruvate levels in schizophrenics compared to controls [32]. NADH dehydrogenase ubiquinone flavoprotein 2 (Ndufv2) is a subunit of mitochondrial complex I and polymorphisms of this are thought to be causative for Parkinson disease [33]. In schizophrenic individuals, a decrease in Ndufv2 was found in both the prefrontal cortex and striatum [34], again in agreement with our PCP results. The mechanism for this effect could be due to the ability of dopamine and glutamate to affect mitochondrial function [34] since PCP is an antagonist at the NMDA receptor among other sites [4]. Therefore, small changes in mitochondrial energy production considerably influence key processes in the cell such as ATP production or the response to oxidative stress [35]. Given a decrease in both Pgam1 and Ndufv2 by PCP in the medial prefrontal cortex in this study, it is clear that the functions of this brain area may be either improved or diminished.

The second class of downregulated proteins includes possible repair mechanisms in response to excessive activity. The enzyme protein-L-isoaspartate (D-aspartate) O-methyltransferase (Pcmt1) forms a part of a repair pathway for aspartyl and asparaginyl groups which are damaged with increasing age [36]. Interestingly, knockdown of Pcmt1 increases phosphorylation of MAP kinases and knockout of this gene affects dendritic arborization [37]. The decrease of Pcmt1 by PCP therefore concurs with the observed upregulation of Mapk1. Proteasome activator subunit 1 (Psme1 or PA28alpha) is a part of the protein degradation process and the production of antigenic peptides [38]. Psme1 also enhances degradation of oxidized proteins and is protective against apoptosis induced by oxidative stress [39]. Therefore, reduction of this by PCP treatment may increase apoptosis susceptibility. The observed decrease of voltage-dependent anion channel 1 (Vdac1) by PCP may be a protective adaptation. Vdac1 is a mitochondrial channel mediating the transport of ions or metabolites between the cytoplasm and mitochondria and has been found to transfer proapoptotic signals [40]. Given the decrease in Psme1 by PCP, a similar decrease in Vdac1 may prevent apoptotic signals and prevent cell death, at least acutely. 

The remaining downregulated proteins are mainly considered as markers for neurodegenerative diseases in that it is more speculative to understand the mechanism or the influence these changes will have on neurotransmission. The epsilon4 allele of apolipoprotein E (apoE) is a known risk factor for late-onset Alzheimer's disease [41]. ApoE is important for the distribution and delivery of lipids in neurons and the clearance of toxic beta-amyloid [42]. Therefore, the downregulation of this may cause amyloid accumulation over time. Bridging integrator 1 (Bin1), also downregulated by PCP in this study, has been associated with Alzheimer's disease together with apoE [43]. Two additional neurodegenerative disease-related proteins were observed in this study. Ubiquitin C-terminal esterase L1 (Uchl1) is a deubiquitinating enzyme although the target proteins of this process are not yet known [44]. Uchl1 is very abundant in neurons and has been associated with Parkinson and Alzheimer's disease [45]. Suppressor of G2 allele of Skp1 (Sugt1) interacts with chaperone proteins like Hsp90 [46]. The Sugt1 protein is abundant in the brain, and cortical neurons expressing Sugt1 are decreased in Alzheimer's disease patients compared to healthy controls [47]. Finally, endoplasmic reticulum protein 29 (ERp29) is involved in the folding of secretory proteins [48] and is abundantly expressed in the brain in all neurons rather than a particular subset [49]. The reduction of ERp29 and Sugt1 by PCP could therefore indirectly suggest the loss of neurons in the medial prefrontal cortex following treatment.

## 5. Limitations

Despite clear advantages with the proteomics approach, there are some limitations in this study. There are limits in terms of the size of protein which can be detected by 2D-DIGE (approximately 200 kDa) such that the majority of large receptor proteins will be missed [12]. This is unfortunate given the interest in neurotransmission and the adaptation of neurotransmitter receptors in various psychiatric disorders. However, receptors are not isolated structures; so one can estimate the effect of the treatment on receptors via changes in messenger or docking proteins. Clearly, a combination of mRNA expression measurement and proteomics would provide a more complete picture. A second limitation concerns the effects of phencyclidine itself. In a previous microdialysis study, we observed an interesting interaction between ethanol and PCP depending on administration order [50]. To simulate executive dysfunction in alcohol-dependent individuals, we used the Cochran and colleagues protocol of 2.58 mg/kg PCP for 5 days [10] but did not see any evidence of PFC dysfunction. With continued optimization and the use of the Y-maze as a behavioural outcome measure, we could detect impairment. However, in another group of animals from our lab, phencyclidine only had a trend to reduction of the percentage of correct alternations [51]. This can be explained by the pattern of locomotion in Figure 1(b). PCP-treated animals moved less than saline-treated controls during the first 5 min of the session, but for the rest of the session the groups were equal. This suggests 5 mg/kg PCP as a threshold dose for affecting Y-maze performance and prefrontal cortex function. The risk of increasing the dose even further, however, is difficult to justify. Higher doses (e.g., 10 mg/kg) have purely toxic effects, while our results indicate that neuroadaptation is a more likely result of 5 mg/kg PCP. A third limitation concerns the small sample size in this study. Given the tendency of screening methods to produce an overwhelming number of hits, we were advised to run a smaller number in order to detect only those proteins most significantly affected by PCP. It is therefore possible that some other interesting protein changes were missed.

## 6. Conclusion 

In a recent review of proteomic studies of postmortem brains of schizophrenics, many proteins were differentially expressed [12]. Comparing this to our study, GNB, Dpysl2, Uchl1, alpha-enolase, and PGAM1 are affected by PCP and in the same direction as reported for schizophrenic brains. This adds to a wealth of evidence that phencyclidine is a valid model of schizophrenia [4]. It also supports the use of the 5 mg/kg dose in Wistar rats and indicates that spontaneous alternation behaviour is a useful outcome measure for future pharmacology studies.

## Figures and Tables

**Table 1:** Summary of the proteins differentially expressed in the medial prefrontal cortex following phencyclidine treatment.

Protein		2D gel		Mass spectrometry			
		Fold change	ANOVA *P* value	MW (kDa)	pI	Score	Peptides in MS/MS
Upregulation by PCP							

Syn1	Synapsin-1	1.3	0.0100	73.9	10	298.0	5
Dpysl3	Dihydropyrimidinase-related protein 3	1.2	0.0271	61.9	6.0	201.5	3
Aco2	Aconitate hydratase	1.1	0.0332	85.4	8.7	224.0	4
Fscn1	Fascin 1	1.2	0.0334	54.5	6.5	299.2	6
Tuba1c	Tubulin alpha-1C chain	1.2	0.0146	49.9	4.8	101.8	2
Mapk1	MAP kinase 1	1.3	0.0192	41.2	6.6	236.9	5

Trend to upregulation							

Gnb4	Guanine nucleotide-binding protein subunit beta-4	1.2	0.0568	37.3	5.7	308.6	4
Capza2	F-actin-capping protein subunit alpha-2	1.2	0.0568	32.9	5.3	117.9	2

Downregulation by PCP							

Bin1	Myc box-dependent-interacting protein 1	1.1	0.0185	64.4	4.8	413.6	7
Dpysl2	Dihydropyrimidinase-related protein 2	1.1	0.0286	62.2	5.9	224.5	6
Sugt1	Suppressor of G2 allele of SKP1 homolog	1.2	0.0389	38.1	5.0	234.6	4
ApoE	Apolipoprotein E	1.3	0.0002	35.7	5.1	260.0	5
Psme1	Proteasome activator complex subunit 1	1.1	0.0182	28.6	5.7	164.8	3
ERp29	Endoplasmic reticulum resident protein 29	1.1	0.0492	28.6	6.3	379.3	5
Pgam1	Phosphoglycerate mutase 1	1.1	0.0479	28.8	6.8	379.3	5
Uchl1	Ubiquitin carboxyl-terminal hydrolase isozyme L1	1.1	0.0313	24.8	5.0	597.4	5
Ndufv2	NADH dehydrogenase ubiquinone flavoprotein 2	1.1	0.0313	27.4	6.3	236.6	3
Pcmt1	Protein-L-isoaspartate (D-aspartate) O-methyltransferase	1.1	0.0068	24.6	7.9	197.1	4
Vdac1	Voltage-dependent anion-selective channel protein 1	1.1	0.0258	30.7	9.2	273.2	4

Trend to downregulation							

EnoA	Alpha-enolase	1.1	0.0996	47.1	6.2	893.8	11
Fh	Fumarate hydratase	1.1	0.0754	54.3	9.7	185.8	2
